# Loading-rate-independent delay of catastrophic avalanches in a bulk metallic glass

**DOI:** 10.1038/srep21967

**Published:** 2016-02-25

**Authors:** S. H. Chen, K. C. Chan, G. Wang, F. F. Wu, L. Xia, J. L. Ren, J. Li, K. A. Dahmen, P. K. Liaw

**Affiliations:** 1 Advanced Manufacturing Technology Research Centre, Department of Industrial and Systems Engineering, The Hong Kong Polytechnic University, Hung Hom, Kowloon, Hong Kong; 2 Laboratory for Microstructures, Shanghai University, Shanghai 200444, China; 3 School of Materials Science and Engineering, Liaoning University of Technology, Jinzhou, 121001, China; 4 School of Mathematics and Statistics, Zhengzhou University, Zhengzhou 450001, China; 5 Department of Physics, University of Illinois, Urbana-Champaign, Illinois 61801, USA; 6 Department of Materials Science and Engineering, The University of Tennessee, Knoxville, TN 37996, USA

## Abstract

The plastic flow of bulk metallic glasses (BMGs) is characterized by intermittent bursts of avalanches, and this trend results in disastrous failures of BMGs. In the present work, a double-side-notched BMG specimen is designed, which exhibits chaotic plastic flows consisting of several catastrophic avalanches under the applied loading. The disastrous shear avalanches have, then, been delayed by forming a stable plastic-flow stage in the specimens with tailored distances between the bottoms of the notches, where the distribution of a complex stress field is acquired. Differing from the conventional compressive testing results, such a delaying process is independent of loading rate. The statistical analysis shows that in the specimens with delayed catastrophic failures, the plastic flow can evolve to a critical dynamics, making the catastrophic failure more predictable than the ones with chaotic plastic flows. The findings are of significance in understanding the plastic-flow mechanisms in BMGs and controlling the avalanches in relating solids.

Catastrophic avalanches are found widely in solids, such as slip events in earthquake faults^1^,^2^, dislocations in crystalline metals^3^,^4^,^5^,^6^,^7^,^8^, and sandpiles^9^, which should be avoided for safety reasons. Recently, a similar phenomenon has been observed in the plastic flow of bulk metallic glasses (BMGs), a class of amorphous alloys without the presence of periodic atomic structures^10^,^11^,^12^,^13^. BMGs are known to have superior properties, as compared to their crystalline counterparts^10^,^11^,^12^,^13^, and have been studied extensively^14^,^15^,^16^,^17^. However, the plastic deformation of BMGs is localized in thin layers of shear bands^18^,^19^,^20^. Without the confinement of crystal lattices and crystalline defects, the rapid propagation of shear bands causes sudden avalanches in BMGs, resulting in brittle separation into two solids parts^10^,^11^,^12^. The statistics of the intermittent avalanches suggest that BMG specimens with minute macroscopic plasticity exhibit chaotic plastic flows^21^. The chaotic behavior in a physical system means that even based on an accurately-controlled initial state, the future of this system is still unpredictable^22^. This trend is an undesirable phenomenon and should be avoided or delayed in practical situations^23^. Therefore, whether the chaotic catastrophic avalanches in BMGs can be changed to the predictable dynamics or delayed to achieve the desirable performance is critical in exploring the application of BMGs, and understanding the deformation nature of solid materials with catastrophic avalanches.

Previous findings have shown that notched BMGs are able to demonstrate delayed catastrophic avalanches under compressive loadings^24^. However, when subjected to tensile loadings, only limited plastic flows consisting of a few flow serrations were achieved^25^,^26^. How to achieve a stable plastic-flow stage and to delay of catastrophic failures under namely tensile loadings are challenging. In the present work, building on a double-side-notched design in tensile BMG specimens where the catastrophic failure occurs through a certain path by initiating a shear avalanche, we delay such a catastrophic fracture process by tailoring distances between the bottoms of the notches in the specimens, where a complex stress field is acquired under the applied loading. A wide range of plastic-flow plateau stage was achieved before catastrophic failures. With tailored notches, the present double-side-notched specimens fracture under mixed mode (I/II) loadings. Based on asymmetric bending tests, Varadarajan *et al*.^27^ and Hassan *et al*.^28^ have reported tremendous increases in fracture toughness of some Zr-based BMGs under mixed mode (I/II) conditions. Tandaiya *et al*.^29^ have also investigated the stress fields of the crack tips under varying mixed mode (I/II) loadings. It has been found that under mixed mode conditions, one part of the notch surface was sharpened while the other part was blunted and the increase in mode II component can enhance the plastic zone sizes^29^. A recent work of Narayan *et al*.^30^ has shown that BMGs are susceptible to a large variability of mode I fracture toughness while the mode II fracture toughness is relatively more stable. Although the fracture behavior of BMGs under varying mode mixity (I/II) has been widely studied^27^,^28^,^29^,^30^,^31^,^32^, the plastic-flow dynamics as well as the delay of the catastrophic avalanches under a complex stress field has yet to be reported. Differing from the strain-rate-dependent plastic flow in compression tests of BMGs with the presence of uniform stress states^33^,^34^,^35^, such a delay of disastrous failure in the present work is found to be independent of loading rate.

## Results

### The delay of the catastrophic avalanches

**Figure 1:**
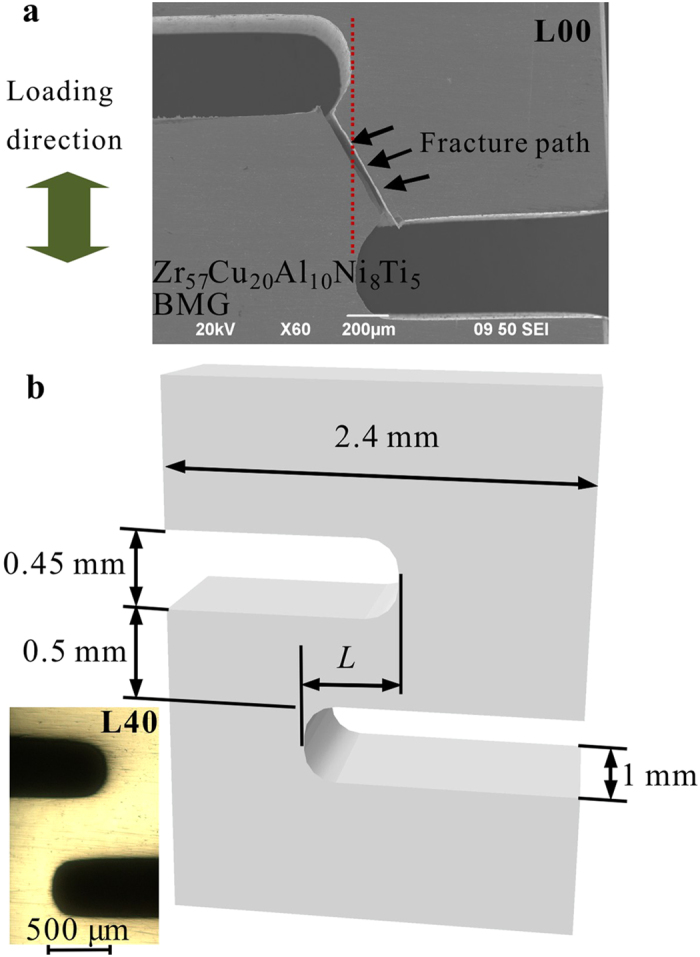
(**a**) Separation of two solid parts of a double-side-notched BMG specimen after tensile testing. (**b**) Schematic diagram of the designed specimens. The specimen in (**a**) with L = 0 mm is noted as L00, and the specimens with L = 0.2 mm and 0.4 mm were labeled as L20 and L40, respectively. The inset optical image in (**b**) shows a prepared L40 specimen.

It is known that nearly all the conventional tensile BMG specimens fracture after the initiation of the first shear band, i.e., no obvious flow serrations have been obtained in the plastic-flow curves^36^. Therefore, to delay the catastrophic avalanches, we first fabricated double-side-notched tensile Zr_57_Cu_20_Al_10_Ni_8_Ti_5_ (atomic percent, at.%) BMG specimens with both notch bottoms aligned along a vertical dashed line (Fig. 1a). When subjected to uniaxial tensile loadings, complex stress fields were achieved in the regions near the notches of the specimens. The stress concentration of both notches ensures the fracture occurring through a path (the formation of a shear band), as shown in Fig. 1a, demonstrating several catastrophic avalanches before the fracture. In order to delay the catastrophic fracture process, we tailor a distance (*L* in Fig. 1b) between the two notch bottoms, as can be seen in the schematic diagram in Fig. 1b. The specimens with *L* = 0 mm (the originally-designed specimen in Fig. 1a), 0.2 mm, and 0.4 mm were noted as L00, L20 and L40 specimens, respectively. Under mixed mode (I/II) loadings, the effective stress intensity factor can be expressed as 
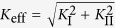
, where *K*_I_ and *K*_II_ are mode I and mode II stress intensity factors respectively^28^,^29^. With *L* = 0 mm, the small bending moment (*M*) acting on the crack tips enables the specimens to fracture at a predominantly mode II loading. When the distance (*L*) increases to 0.2 mm and 0.4 mm, the bending moment (M) also increases, resulting in the increase of the mode I component *K*_I_. Thus, the mode mixity^37^ *m*^e^ = *K*_I_/*K*_II_ increases as the distance *L* increases. Since in this work we mainly focus on the plastic deformation behavior (plastic-flow dynamics) of the regions between two notches and the facture of BMGs under varying mode mixity has been documented^27^,^28^,^29^,^30^,^31^,^32^, the effect of varying mode mixity (I/II) on the fracture of the present specimens is not discussed in detail here.

**Figure 2:**
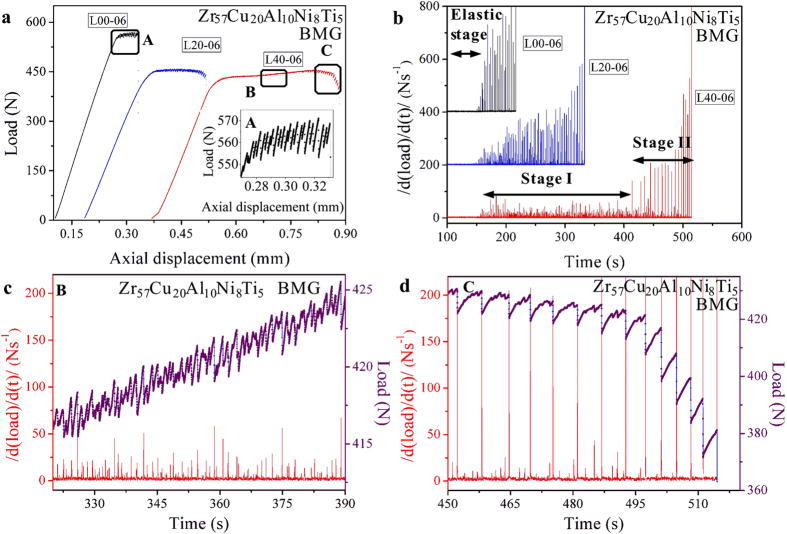
(**a**) Tensile-testing results (at a loading rate of 0.06 mm/min.) showing the delay of the catastrophic load drops in the notched Zr_57_Cu_20_Al_10_Ni_8_Ti_5_ BMG specimens, and the inset presents the magnified serrated flow of rectangle A. (**b**) Corresponding |d(load)/d(t)| vs. time relations indicating the delay of the avalanches. (**c,d**) Close observation of the serrated avalanches and the corresponding |d(load)/d(t)| vs. time relations of rectangles B and C, respectively, in (**a**).

The typical load-displacement curves of the notched BMG specimens (at the loading rate of 0.06 mm/min.) are given in Fig. 2a. The L00 specimen exhibits a serrated flow consisting of 28 load drops (shear avalanches) with load magnitudes ranging from 0.15 N to 37.66 N (The load drops with a size smaller than 0.15 N are caused by the vibration of the testing machine, and are neglected here). The bursts of load drops correlate with the formation of shear bands and the intermittent sliding in the BMG specimen^38^. It can be found that the size of load drops increases during the loading process (Fig. 2a inset), which suggests that the load drops could be impeded initially, and with the increase of the axial displacement, the propagation of shear bands cannot be impeded any further. The rapid propagation of a shear band creates the separation between two solid parts, resulting in the final catastrophic failure of the specimen^18^,^19^.

**Figure 3:**
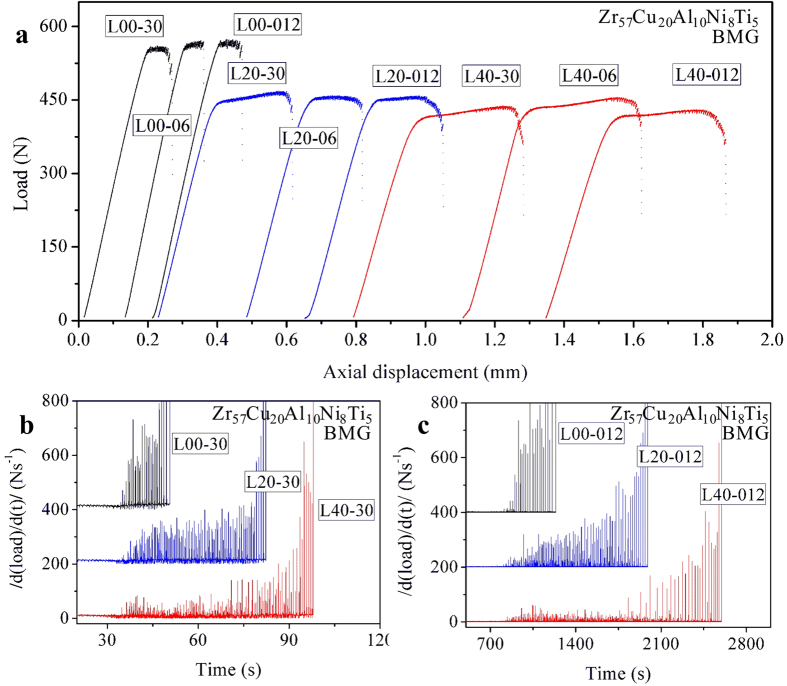
(**a**) Load-axial displacement curves of the specimens under various loading rates of 0.3 mm/min. (−30), 0.06 mm/min. (−06), and 0.012 mm/min. (−012). (**b,c**) are the corresponding |d(load)/d(t)| vs. time relations under loading rates of 0.3 mm/min. and 0.012 mm/min., respectively.

With increasing *L* values (Fig. 1b), a plastic-flow plateau stage appears before catastrophic failures in the L40 specimens (Fig. 2a). To characterize the intermittent bursts in the plastic flow of these specimens, corresponding |d(load)/d(t)| vs. time relations were plotted in Fig. 2b. It can be seen that in all the elastic stages of the specimens, the sizes of the |d(load)/d(t)| values are small enough that during these stages, the perturbations of the loads can be neglected. This trend is true for the fact that the small perturbations in these stages are caused by the vibration of the testing machine. At the plastic-flow stages, the L00 specimens show large magnitudes of the |d(load)/d(t)| values, indicating the large avalanches in this specimen. The L20 specimen shows a delaying trend of the catastrophic avalanches, while the L40 specimen obviously presents a stage with relatively-smaller magnitudes of avalanches (stage I) before the large avalanches (stage II). Figure 2c,d show the magnified plastic-flow serrations as well as corresponding |d(load)/d(t)| vs. time relations in stages I and II, respectively. At the relatively-stable plastic-flow stage (stage I), the plastic flow has a larger number of bursts but with smaller burst sizes, as compared to the large plastic-flow stage (stage II). The first stage of plastic flows consists of small sizes of load drops, less than 5 N (Fig. 2c), while the second stage of deformation has load-drop sizes reaching 20 N (Fig. 2d), similar to the L00 specimen. Previous studies on the plastic flow of BMGs under compression tests have shown that the criticality of the bursts of load drops (shear avalanches) is significantly influenced by the applied loading rate^34^,^35^. However, the results in the present work demonstrate that the delay of catastrophic avalanches also occurs at loading rates of 0.3 mm/min. and 0.012 mm/min. (Fig. 3). It suggests that the delay of catastrophic avalanches in the double-side-notched BMG specimens might be independent of the loading rate.

### Statistics analysis

**Figure 4:**
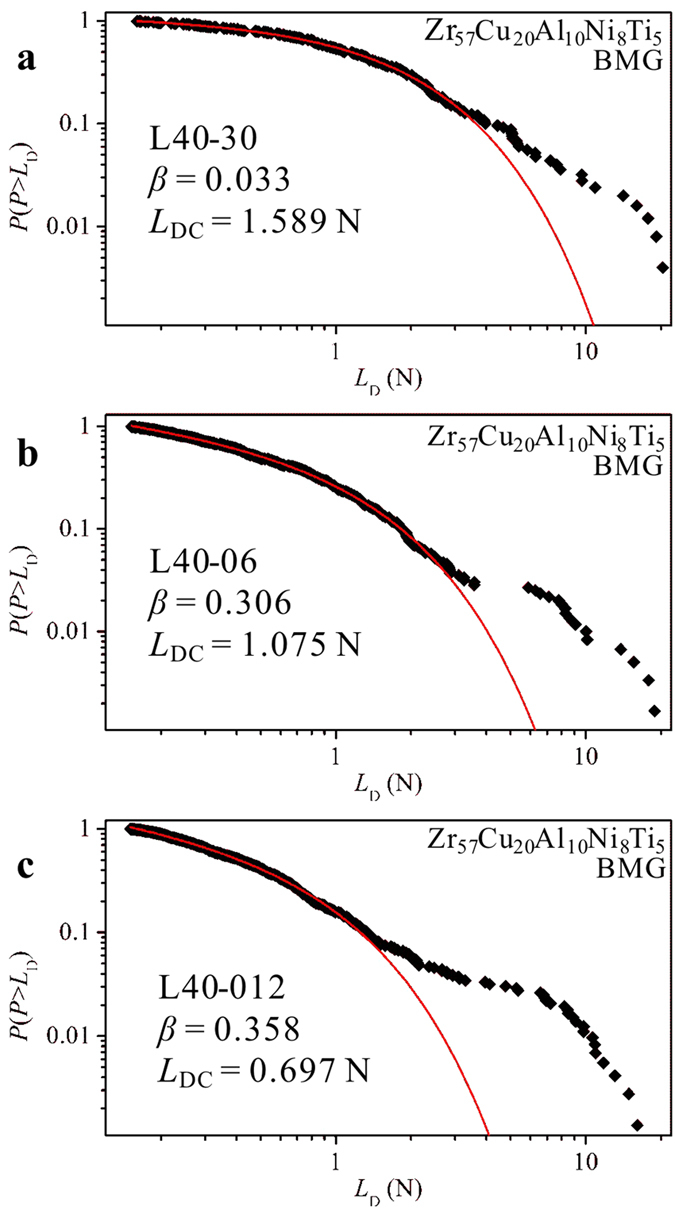
The cumulative probability distributions (the scattered points) of the bursts of load drops in the L40 specimen at loading rates of 0.3 mm/min. (**a**), 0.06 mm/min. (**b**), and 0.012 mm/min. (**c**). The solid red lines are the corresponding fitting curves acquired based on Equation (1).

Taken the plastic-flow stage consisting of shear avalanches (bursts of load drops) as a physical system, the cumulative probability distributions of the bursts of load drops, i.e., the percentage of the number of load drops larger than a given load drop, *L*_D_, *P*(*P* > *L*_D_), is calculated as shown in Fig. 4. Using a universal power-law scaling function, the cumulative probability distributions of *P*(*P* > *L*_D_) have been modeled as


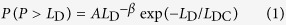


where *A* is a normalized constant, *β* is a scaling exponent, and *L*_DC_ is the cut-off load drop magnitude^39^. The fitting parameters, *β* and *L*_DC_, are given in Fig. 4. It can be found that load drops with smaller amplitudes are able to follow a power-law distribution, while the load drops with larger amplitudes have an exponential decay. The power-law distributions suggest that the L40 specimens might exhibit the critical scaling behavior^6^,^40^. Although the cut-off load drop magnitude (*L*_DC_) decreases, as the loading rate decreases, the presence of the delaying trend of the catastrophic failures and the critical scaling behavior in the L40 specimens is independent of loading rate.

**Figure 5:**
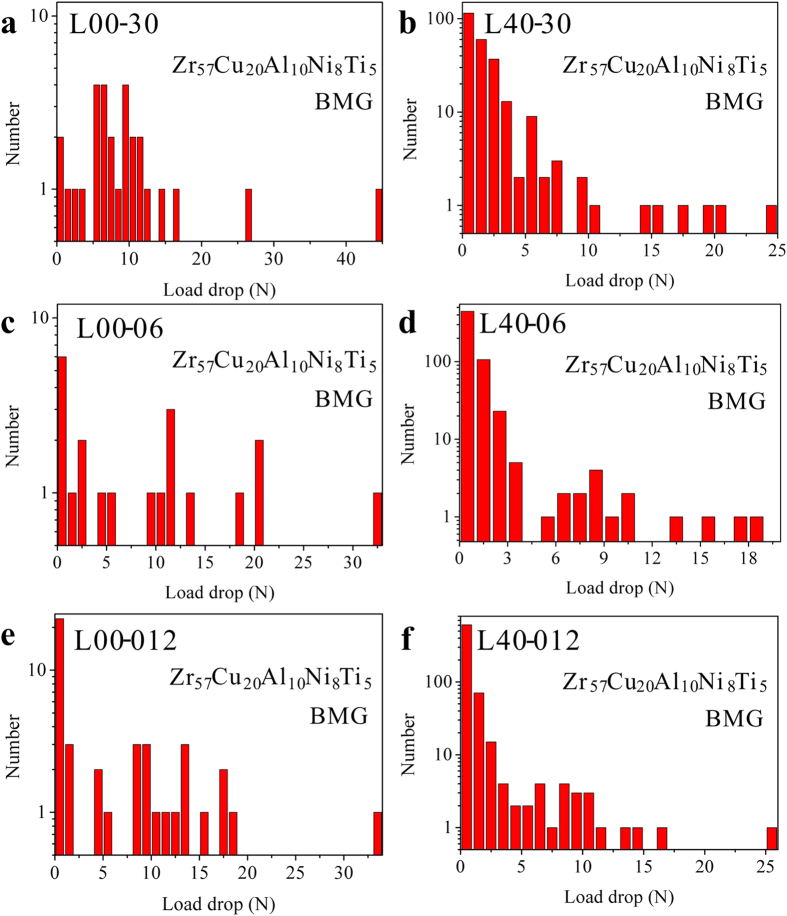
Statistical results of the distribution of load drops of the L00 (**a,c,e**) and L40 (**b,d,f**) specimens at loading rates of 0.3 mm/min. (**a,b**) 0.06 mm/min. (**c,d**) and 0.012 mm/min. (**e,f**).

To further verify the speculation, the statistics of the bursts of the shear avalanches (the sizes of the load drops) of L40 specimen is shown in Fig. 5. It is obvious to find that the L00 specimens (Fig. 5a,c,e) have scattered load drops with various amplitudes, suggesting a chaotic behavior during the plastic-flow stage^21^,^41^. While the L40 specimens (Fig. 5b,d,f) have larger numbers of load drops with amplitudes smaller than 5 N. The statistic results are consistent with the findings of the serrated plastic flow in Figs 2 and 3 that a large number of shear avalanches in Stage I has smaller amplitudes smaller than 5 N. The distribution of the bursts of the load drops less than 5 N were modeled, using a power-law scaling expressed as


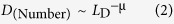


**Figure 6:**
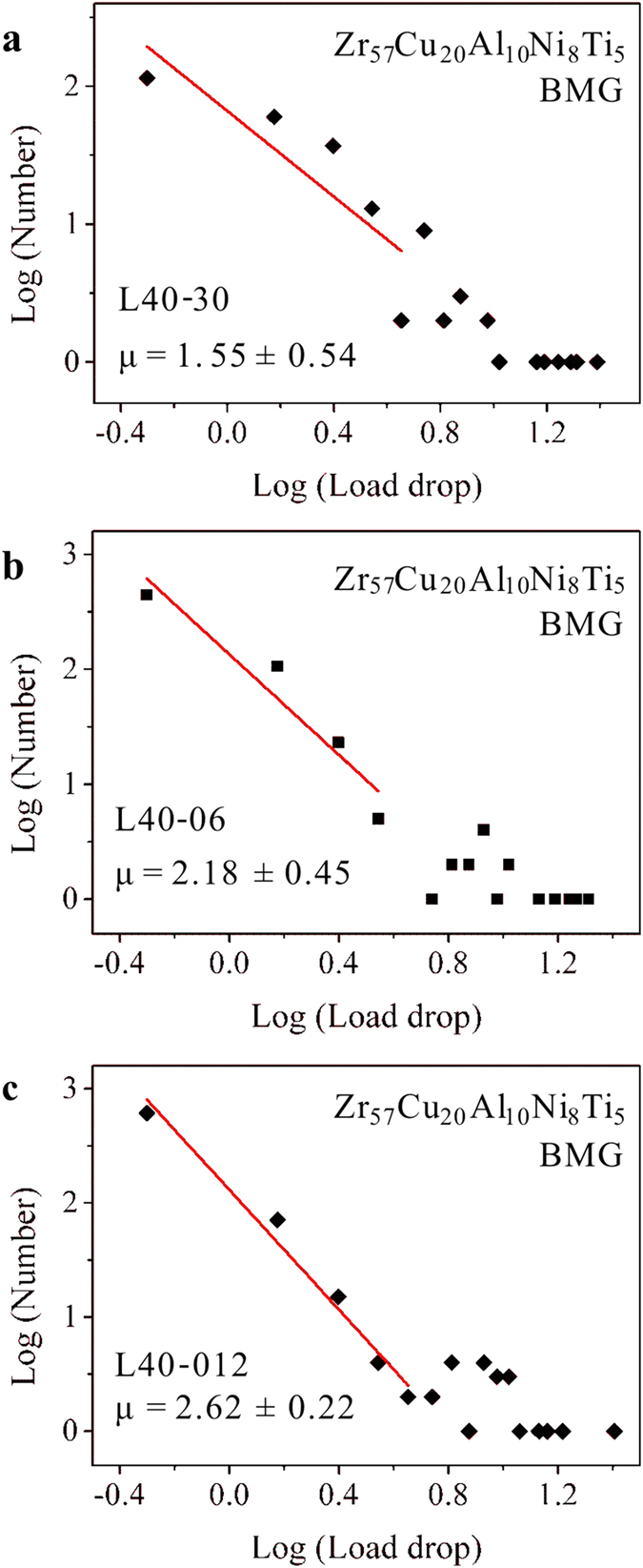
Linear fit of the power-law distributions of the L40 specimens at loading rates of 0.3 mm/min. (**a**), 0.06 mm/min. (**b**), and 0.012 mm/min. (**c**), respectively.

The fitting results are shown in Fig. 6. It can be found that the distribution of the bursts of the load drops with smaller magnitudes (less than 5 N) follows a power law. While the load drops with magnitudes larger than 5 N display non-ordered distributions. The well-modeled power-law distributions further validate that the intermittent plastic flow of the L40 specimen operates in a near-critical state, i.e., exhibiting critical-scaling behavior^6^,^23^. This trend implies that the bursts of load drops (corresponding to the initiation of shear bands) are affected by previous flow serrations, and the triggering of the bursts of the load drops during the plastic-flow stage might be inherently correlated. By carefully-examining the flow serrations in the BMG specimens, it can be found that in the L00 specimens (Fig. 2a inset), the burst of large load drops (larger than 5 N) is also followed by the occurrence of large load drops within the several subsequent flow serrations. While in the first-stage plastic flow of the L40 specimens (Fig. 2c), the burst of small load drops (smaller than 5 N) tends to trigger the formation of also small load drops, resulting in the critical plastic-flow dynamics. Thus, the observation of the burst of a load drop equal or larger than the critical value, i.e., 5 N in the present work, might be regarded as an indicator for the beginning of a chaotic plastic-flow stage before the final catastrophic failure of the specimen. The observation of the critical dynamics in the L40 specimens makes the catastrophic shear avalanches in BMGs become more predictable, as compared with the chaotic plastic flow in the L00 specimens.

### Shear-band initiation and propagation

**Figure 7:**
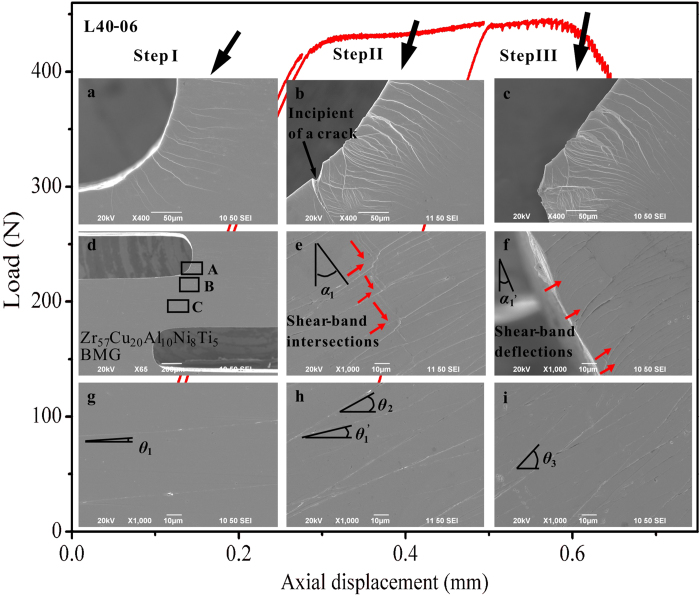
The three-step evolution in an L40 specimen (at a loading rate of 0.06 mm/min.), and the insets are the corresponding SEM micrographs, where (**a,d,g**), (**b,e,h**), and (**c,f,i**) are the SEM images at steps I, II, and III, respectively. The inset image (**d**) shows an overview of the notched specimen, (**a–c**) are the magnified images of region **A** in (**d**–**f**) are the magnified images of region **B**, and (**g–i**) the magnified images of region **C**.

Since the bursts of load drops are correlated with the formation of shear bands^38^, the formation and propagation of shear bands in the BMG specimens, under a typical loading rate of 0.06 mm/min., are carefully inspected. As shown in Fig. 7, the evolution of shear bands in the L40 specimen was examined by dividing the tensile test into three steps. After each step of the test, the formation of shear bands were inspected, using scanning-electron microscopy (SEM). After the deformation at step I, some shear bands initiate at two regions: region **A** at the edge of the notch (Fig. 7a,d) and region **C** at the middle of the specimen (Fig. 7d,g). The formation of shear bands in region **A** results from the stress concentration at the notches. In region **C**, the shear bands deviated the transverse direction at an angle of *θ*_1_, as indicated in Fig. 7g (for simplicity, we name these shear bands as *θ*-shear bands). This trend implies that some localized plastic flow occurs along the roughly**-**transverse direction of the specimen.

When the plastic flow undergone a stable plateau (step II), the evolution of shear bands is shown in Fig. 7b,e,h. Firstly, at the edge of the notch (region **A**, as shown in Fig. 7b), more shear bands are initiated, and the existing shear bands at step I are further extended, including some bifurcations and intersections. Moreover, the incipient of a crack has been observed in this region, as indicated in Fig. 7b. Secondly, the shear bands deviated from the vertical direction at an angle of *α*_1_ in Fig. 7a, which are classified as *α*-shear bands in this work, have propagated to the inner part of the specimen, i.e., region **B** in Fig. 7e. It can been seen that the propagation of the *α*-shear bands have been blocked by the several *θ*-shear bands, including many intersections (indicated by the red arrows in Fig. 7e). Each intersection event causes the *α*-shear bands to shift at a small distance along the direction of the *θ*-shear bands. Thirdly, at region **C** (Fig. 7h), the middle of the specimen, the existing shear bands in Fig. 7g further deviated from the transverse direction at an angle of *θ*_1_′. On the other hand, the formation of some new shear bands at an angle of *θ*_2_ is observed. This trend indicates that during the stable plastic-flow stage, the whole specimen were shear-deformed at an angle of the magnitude of (*θ*_1_′ − *θ*_1_), and more shear flows occurred.

Figure 7c,f,i show the distribution of shear bands after fracture (step III). The crack initiated at step II (Fig. 7b) has propagated throughout the whole specimen, causing the catastrophic failure of the specimen. The fracture plane deviated from the vertical direction at an angle of α_1_′. Near the fractured plane (Fig. 7f), it can be found that the *θ*-shear bands have been deflected significantly, indicating the highly-blocking effect before the catastrophic failure. In the middle of the specimen (Fig. 7i), besides the increase of the deviation angles (*θ*_1_′ and *θ*_2_), more shear bands were formed at larger deviation angles (*θ*_3_), further verifying the macroscopic shearing of the specimens.

**Figure 8:**
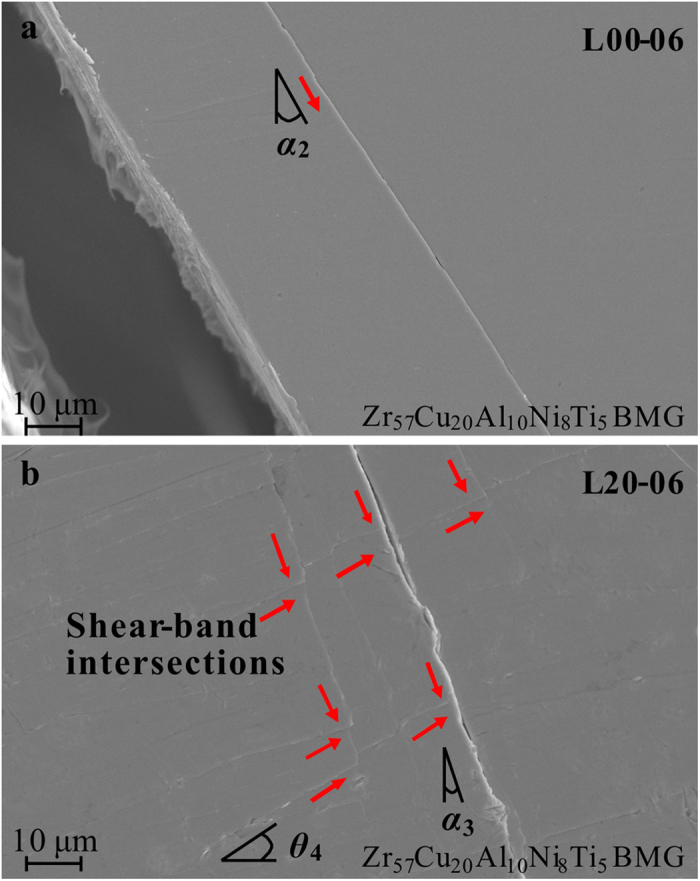
SEM images showing the blockage of the α-shear bands by the formation of *θ*-shear bands in the fractured L00 and L20 specimens (at a loading rate of 0.06 mm/min.).

The formation of shear bands during the whole deformation and fracture process in the L40 specimen can be categorized into two classes. The *α*-shear bands initiate from the edge of the notches, and propagate to the roughly-vertical directions at a deviation angle of *α*. The other class of shear bands (*θ*-shear bands) initiate from the inner side of the specimen, deviated from the transverse direction at an angle of *θ*. It can be speculated that the initiation and propagation of *α*-shear bands correlate with the catastrophic shear avalanches with relative larger load-drop magnitudes, while the formation of the *θ*-shear bands hinders the propagation of the *α*-shear bands, delaying the catastrophic avalanches by forming a stable plastic flow. This prediction can be further verified by the SEM observations of the L00 and L20 specimens. In Fig. 8a, the fractured L00 specimen only shows *α*-shear bands without the observation of *θ*-shear bands. In Fig. 8b, the blockage of the *α*-shear bands was presented in the L20 specimen (indicated by the red arrows), which shows a slight delay of the catastrophic avalanches. The significant blockage of the *α*-shear bands in the L40 specimen has been shown in the SEM observations in Fig. 7e. The formation of a large number of *θ*-shear bands plays a significant role in blocking the rapid propagation of *α*-shear bands to avoid the rapid fracture of the specimen.

## Discussion

**Figure 9:**
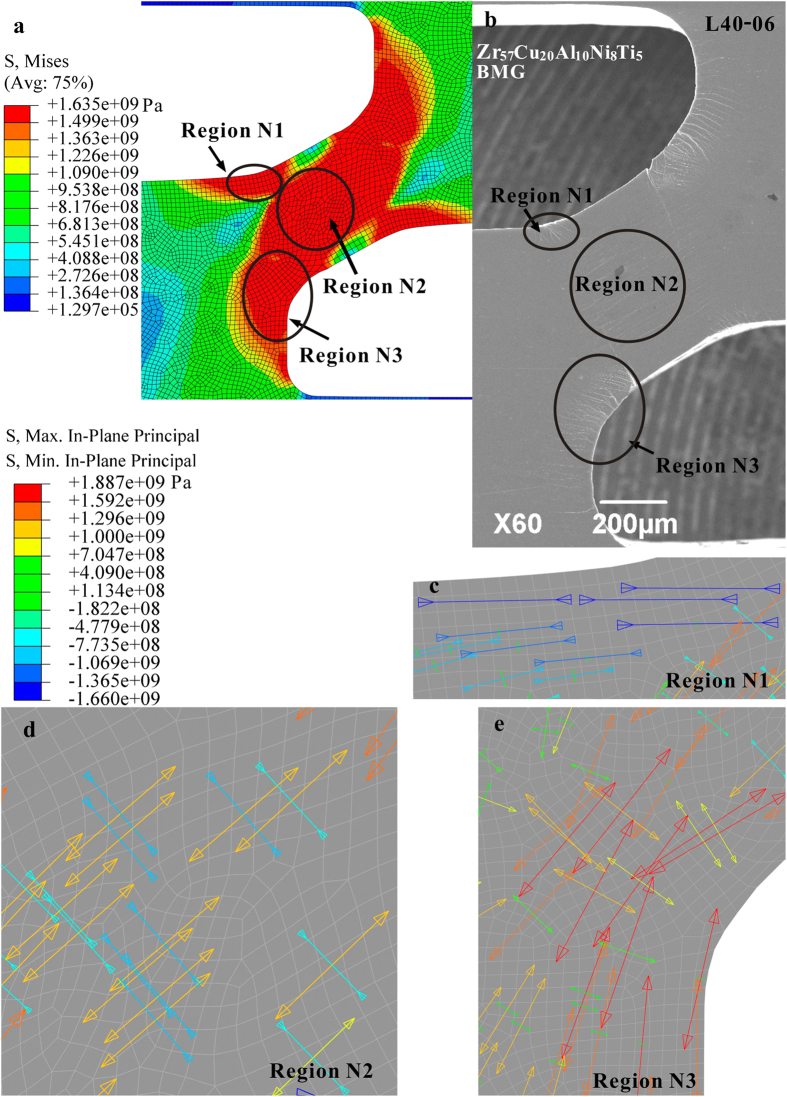
(**a**) shows the FEM results of the Mises stress distribution of the L40 specimen at step II in Fig. 7, and (**b**) is the corresponding SEM image. (**c–e**) present the magnified principal stress distributions in the corresponding regions **N1**, **N2**, and **N3**, respectively, where the arrows show the direction of the principal stresses and arrows sizes and colors indicate the proportional values of stresses.

In the physical system of the plastic flow of BMGs, the delay of the shear avalanches correlates with the confinement of the propagation of shear bands and cracks. It has been reported that the presence of a complex stress field plays a significant role in influencing the catastrophic fracture process of BMGs^24^,^25^,^26^,^42^,^43^,^44^,^45^,^46^,^47^. Figure 9a shows the Mises distribution of the L40 specimen through finite-element modelling, where shear bands nucleate at regions **N1**, **N2**, and **N3**. It seems that the specimen could fracture rapidly, since the yielded region transverses the whole specimen. However, on the SEM image (Fig. 9b) of the specimen, although we do observe many shear bands in these regions, the shear bands distribute at various directions and densities. It can be seen that regions **N1** and **N3** have *α*-shear bands, while region **N2** has *θ*-shear bands, and region **N3** has the densest shear-band distribution, where cracks initiate (the detail information of the formation of shear bands and cracks in regions **N2** and **N3** is shown in Fig. 7b,e,h).

**Figure 10:**
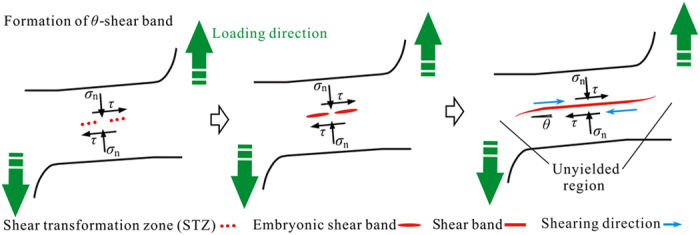
The schematic diagram showing the formation of *θ*-shear bands in region N2 (region C in Fig. 7).

Further examining the maximum and minimum principal stress distributions (Fig. 9c–e) gives some evidences for understanding the shear-band patterns. According to the FEM results (Fig. 9c–e), the arrows show the principal stress directions, where the arrows sizes and colors indicate the proportional values of stresses. In region **N1** (Fig. 9c), this part of the BMG undergoes a compressive stress state, which explains the results that this region has the smallest density of shear bands, and the shear bands in this region will not evolve to cracks to cause catastrophic failures. In region **N3** (Fig. 9e), this part of the BMG has both tensile and compressive stresses. But the magnitudes of the tensile stresses are much greater than the compressive ones. With the largest maximum principal stress, cracks initiate from the edges in this region, and propagates towards the direction with smaller principal stresses. In region **N2** (Fig. 9d), it is interesting to find that this region has both tensile and compressive stresses with relatively-same orders of magnitudes. Greer *et al*.^48^ have summarized three possible scenarios for the formation of shear bands in BMGs, where the inevitable casting defects, such as voids and surface notches, and the defect-induced stress concentrations play significant roles. Johnson and Samwer^49^ have shown that the applied stress is also important for the activation of a shear transformation zone (STZ). For θ-shear bands in region **N2** (region **C** in Fig. 7), the shear band can be nucleated by a combining effect of both casting defects and the applied stress. As shown in a schematic diagram in Fig. 10, under applied loading, STZs will be firstly activated at the sites of casting defects, such as the nano voids^48^,^49^. Embryonic shear bands will then be formed by activating a group of STZs^48^. When the size of the embryonic shear band reaches a critical size, the embryonic shear band will evolve to a shear band^50^ and propagate at an angle *θ*. The propagation of the shear band will then be blocked by the unyielded region which is subject to a lower stress level. To fracture, the crack initiated from region **N3** must cross over region **N2**. Thus, the presence of region **N2** is critical for driving the system to evolve to a critical state (Figs 2 and 3).

The delay mechanisms in this BMG specimen can, then, be easily understood: the shear bands and cracks, initiated from region **N3**, correlate with the catastrophic shear avalanches with large magnitudes. Without region **N2** in the L00 specimen, the propagation of shear bands/cracks transverses the specimen rapidly. While in the L40 specimen, the presence of region **N2** drives the formation of a large number of *θ*-shear bands and blocks the propagation of the *α*-shear bands/cracks, introducing a large number of small shear avalanches, which postpones the large avalanches to result in a stable plastic-flow plateau.

**Figure 11:**
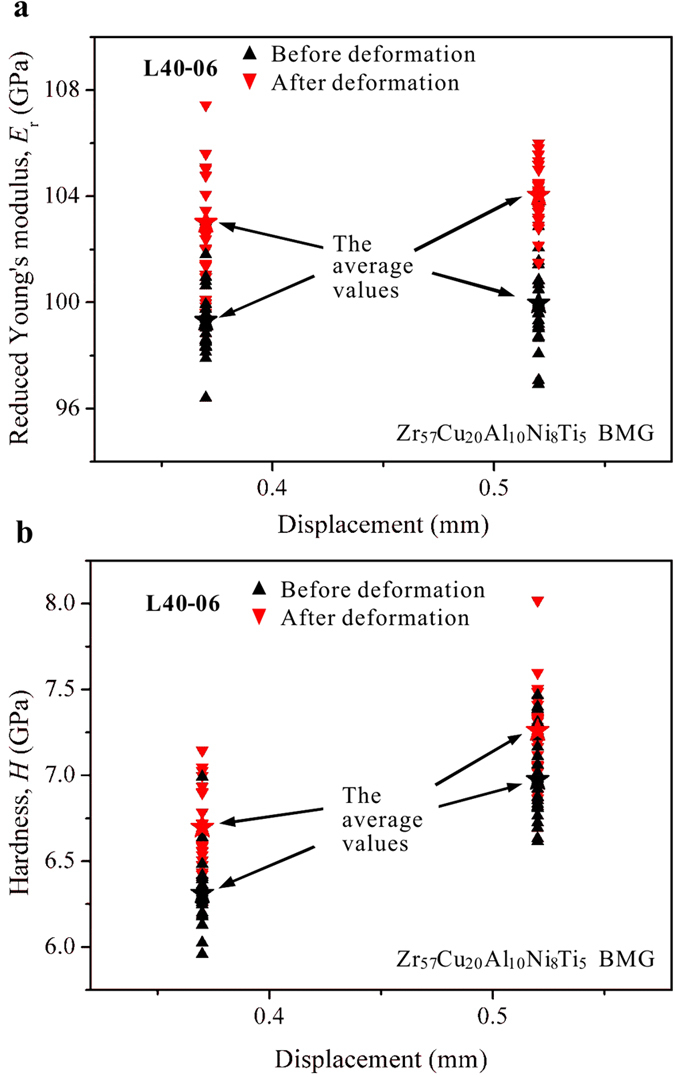
Nano-indentation testing results showing the changes of the reduced moduli (**a**) and hardness (**b**) of two L40 specimens (at a loading rate of 0.06 mm/min.) after axial displacements at 0.37 mm and 0.52 mm (at fracture), respectively.

The nanoindentation testing results in region **N2** are shown in Fig. 11. It can be seen that after a steady plastic-flow stage (i.e., the plastic-flow plateau in Fig. 2a), the reduced Young’s modulus (*E*_r_) and hardness (*H*) increased. The results are different from the usual “work-softening” nature in BMGs that the formation of shear bands will generate more free volume, which will cause decrease in Young’s modulus and hardness^11^,^19^,^51^,^52^,^53^,^54^. This phenomenon might result from the complex stress field in region **N2**. It has been reported that, in a BMG with similar composition (Zr_64.13_Cu_15.75_Ni_10.12_Al_10_, at.%), the multi-axial stress states can cause strain hardening (increased hardness and failure stress) through structural disordering or annihilation of free volume^44^. The increased reduced Young’s moduli and hardness may also contribute to the stable plastic-flow stage. For a crack to cross over this region (**N2**), the critical stress intensity factor of the BMG can be expressed as *K*_IC_ = (CTOD*mσ*_y_*E*)^1/2^, where CTOD is the crack-tip open displacement, *m* is a dimensional constant, *σ*_y_ is the yield stress, and *E* the Young’s modulus^55^,^56^. With increased modulus, the *K*_IC_ value also increases, resulting in more energy being dissipated for a crack to transverse this region. This trend further verifies the inhibition of the crack propagation in region **N2** to delay the catastrophic shear avalanches.

BMGs are known to have catastrophic failures under uniaxial tensile loading. Although much effort has been made to delay such catastrophic failures, most of the research focused on developing composite microstructures by creating crystalline phases^57^,^58^,^59^,^60^. The present work demonstrates that the catastrophic avalanches in BMGs can be delayed by creating complex stress fields through tailoring the specimen geometries (notches). Some efforts have been made to understand the plastic deformation behavior of notched BMG specimens under tensile loading^25^,^26^, however, only limited numbers of load drops were observed and the catastrophic avalanches remained unavoidable. In the present double-side-notched specimens, the resultant complex stress field under applied loading can result in more plastic deformation in the stress-concentrated region to form a stable plastic-flow stage before the catastrophic failures, which can evolve into a critical state. Some studies have also reported that in the compression tests of BMGs, the BMG specimens with large macroscopic plasticity exhibit power-law scaling behavior^61^ or even some characteristics of the self-organized criticality (SOC) behavior^21^,^62^. However, such characteristics of the critical behavior highly depend on the applied strain rate^34^,^35^. While the present notched geometry-meditated critical behavior in the plastic flow of BMGs does not changes, as the loading rate varies from 0.3 mm/min. to 0.012 mm/min. This phenomenon is different from the conventional compressive testing results of BMGs where the whole specimen is involved in the deformation process. The burst of shear bands is associated with the initiating of STZs, while the potential energy barrier for activating an STZ is determined by both the internal physical properties and the external applied shear stress^48^,^49^. With relatively uniform stress state (compressive) in conventional compression tests, the activation of STZs is mainly governed by the internal physical nature of the material. The initiation and propagation behavior of shear bands will therefore be changed as the spatial and temporal conditions change. It is well known that the plastic flow in BMGs is path-dependent and sensitive to the applied strain rates^36^,^63^,^64^,^65^. However, in the present double-side notched specimens, the FEM results (Fig. 9) show that yielding starts from the stress-concentrated regions and evolves to the regions with lower stress level (unyielded regions). As compared with conventional compressive tests, the deformation mechanisms are different, and the evolution of the yielded regions in these notched specimens is less sensitive to the loading rate. The effect of loading rate on the plastic flow in these notched specimens is therefore less significant. The findings show that, besides the tuning of internal physical properties, the tailoring of complex stress fields could also play an important role in arresting the catastrophic shear avalanches in BMGs, and making the disastrous failures more predictable. Moreover, the delayed catastrophic avalanches in BMGs suggest that the avalanches in some relating solids could also be postponed, if the appropriate external drive is applied.

## Methods

The as-cast Zr_57_Cu_20_Al_10_Ni_8_Ti_5_ (atomic percent, at.%) BMG rods with 3 mm in diameter were fabricated, using copper-mold casting. The L00, L20, and L40 specimens (Fig. 1) were fabricated from the as-cast rods, and the notches were cut, using a diamond saw. The surfaces of the specimens were polished, employing abrasive papers with grits from 150 to 2,000. Tensile tests of the designed specimens were performed on an Instron 5565 electromechanical materials testing machine at constant cross-head displacement rates of 0.3 mm/min., 0.06 mm/min., and 0.012 mm/min. The data were recorded at 100 points per second. The magnified data of the elastic flow of the specimens indicate that the small avalanches with sizes less than 0.15 N was caused by the vibration of the testing machine. Thus, the avalanches with sizes smaller than 0.15 N were ignored in the statistical analysis. The surfaces of the specimens were inspected, using a Jeol JSM-6490 scanning electron microscope. The nanoindentation tests was conducted on a TRIBO INDENTER (Hysitron CO. LTD) machine with a Berkovich diamond tip. A pure aluminium sample was used as a standard sample for the initial calibration. The maximum load was supposed to be 10 mN at a loading rate of 2 mN/s. At each loading rate, ten nanoindentation tests were carried out to exclude the occasional results. The peak load was held constant for 5 s. The unloading rate was 2 mN/s. The elastic modulus and the hardness value were measured from the load-displacement curve in the unloading stage. Finite-element-modelling (FEM) of the distribution of the Mises and in-plane stress in the L40 specimen was conducted, using the commercial ABAQUS software, based on an ideal elastic–plastic constitutive model^24^,^66^. The input material parameters of the Poison’s ratio, Young’s modulus, and yield stress were 0.36^67^, 82 GPa^67^, and 1.635 GPa^45^ respectively.

## Additional Information

**How to cite this article**: Chen, S. H. *et al*. Loading-rate-independent delay of catastrophic avalanches in a bulk metallic glass. *Sci. Rep*. **6**, 21967; doi: 10.1038/srep21967 (2016).
